# Non-alcoholic fatty liver disease in a rural, physically active, low income population in Sri Lanka

**DOI:** 10.1186/1756-0500-4-513

**Published:** 2011-11-24

**Authors:** M Janani Pinidiyapathirage, Anuradha S Dassanayake, Shaman Rajindrajith, Udaya Kalubowila, Norihiro Kato, A Rajitha Wickremasinghe, H Janaka de Silva

**Affiliations:** 1 Faculty of Medicine, University of Kelaniya, P O Box 6, Ragama 11010, Sri Lanka; 2 Department of Gene Diagnostics and Therapeutics, Research Institute, National Center for Global Health and Medicine, 1-21-1 Toyama, Shinjuku-ku, Tokyo 162-8655, Japan

## Abstract

**Background:**

Non-alcoholic fatty liver disease (NAFLD) is recognized as a metabolic disorder largely seen in urbanized populations. The purpose of this study was to assess prevalence and risk factors for NAFLD in a rural, physically active, economically deprived population in Sri Lanka.

**Methods:**

By visiting individual households in the community, 35-64 year old adults resident in two selected estates in the Nuwara Eliya District of Sri Lanka, were invited to participate in the study. Blood pressure and anthropometric measurements were made on all participants. Blood samples were obtained for the assay of fasting glucose, serum lipids, serum insulin and alanine aminotransferase. NAFLD was diagnosed on established ultrasound criteria for fatty liver in the absence of hepatitis B and C markers and high alcohol consumption.

**Results:**

Of those invited, 403 (65%) participated in the study. Almost all participants were either Indian or Sri Lankan Tamils and 53% were females. Prevalence of NAFLD was 18% in this population. Twice as many males were diagnosed as having NAFLD compared to females. Male sex, high BMI, high waist circumference, high diastolic blood pressure and high plasma glucose levels were significant predictors of NAFLD.

**Conclusion:**

Nearly one in five people in this predominantly Indian Tamil, rural, physically active, economically deprived population had NAFLD. The condition was associated with constituent features of the metabolic syndrome. These results support studies reporting ethnic variations in disease susceptibility and suggest that genetic factors may also play a role in determining disease risk.

## Background

Non-alcoholic fatty liver disease (NAFLD) is now recognized as the most common chronic liver condition in most regions worldwide. The majority of patients with this condition generally have simple hepatic steatosis with no or mild nonspecific inflammation. However, more active forms of the condition that include steatohepatitis can have significant clinical consequences related to the development of cirrhosis and its complications or co-morbid cardiovascular disease [1].

The prevalence of NAFLD has accelerated in the last few decades, paralleling the substantial increase in rates of overweight and obese people in the general population [1]. NAFLD was conventionally recognized as a metabolic disorder largely confined to residents of affluent industrialized Western countries [2]. The estimated prevalence ranges between 2.8% and 46% in the general population in these countries depending on the screening test used [2]. However, it is now clear that NAFLD is no longer a disease confined to affluent Western societies. Emergence of NAFLD as a significant public health problem in Asian populations, especially in regions with significant industrialization or urbanization, has been reported in several recent studies [3,4]. A recent population based study from Sri Lanka reported a NAFLD prevalence of 32.6% in an urban predominantly sedentary population [5]; Sri Lanka is currently experiencing a surge in non--communicable diseases as a result of the epidemiologic and demographic transitions that have interacted with lifestyle changes. However, data on NAFLD prevalence in less urbanized, socioeconomically underprivileged populations in Sri Lanka, and in most Asian countries, is not available. This study aims to determine the prevalence of NAFLD and its risk factors in the estate population of Sri Lanka, a physically active, low socio-economic population group.

## Methods

This study was part of a large community-based investigation on non-communicable diseases: the Ragama Health Study (RHS) [5]. The RHS is a collaborative study between the International Medical Centre of Japan (IMCJ) and the Faculty of Medicine, University of Kelaniya, Sri Lanka. Ethical approval for the study was obtained from the Ethical Review Committees of the Faculty of Medicine, University of Kelaniya and the IMCJ.

Data from the estate component of the study are presented here. All participants in this study were recruited from Nuwara Eliya; located approximately 180 kms from Colombo, the capital city. Nuwara Eliya District has the largest estate population (54% of the district's population) in the country. The estate sector is defined as plantations of 20 acres or more in extent on which there are 10 or more resident labourers [6]. According to the Household Income and Expenditure Survey - 2006/07 [7], both median household income and expenditure in the estate sector are below that of the national figures. Percentage with education upto Grade 10 in the estate sector is only 3% as compared to 15% nationally. In the estates, only about 10% own a vehicle (at least a bicycle) as compared to 56% nationally and over 80% of the estate residents report walking as the main mode of travel.

### Study population

The study population consisted of 620 adults in 35-64 year age group resident in two selected estates in the Nuwara Eliya District. The estates were selected on feasibility for future follow up and availability of sufficient infrastructure facilities to conduct a risk assessment study.

### Sampling and subject recruitment

All individuals in the 35-64 year age group residing in the two estates were invited to participate in the study during the period March-June 2008. The list of employees maintained by the estate management was used as the sampling frame. A doctor accompanied by health personnel from the estate medical center visited individual households in the community and invited people to participate. Participants were requested to present after a 12 h fast with any available health records. Written consent was given by all adults agreeing to participate in the study.

### Measurements

Participants were subjected to an interview, ultrasonographic examination of the liver, anthropometric and blood pressure measurements and collection of blood samples. They were interviewed by trained community volunteers to obtain information on socio-demographic variables and lifestyle habits. Details regarding the type, and amount of alcohol consumed and duration of drinking were also obtained. All past medical records of the subjects were analyzed and recorded. Normal cut-off values were based on the revised Adult Treatment Panel III (ATP III) criteria for metabolic syndrome for Asians [8]. A 10 ml sample of venous blood was obtained from each subject. This was used to determine fasting glucose, serum lipids, serum insulin and serum alanine aminotransferase activity (ALT). All subjects underwent ultrasonography of the liver with a 8-MHz probe (Toshiba Ultrasound Diagnostic Systems SSA-51 OA, Toshiba Medical Systems Corporation, Otawara-City, Tochigi-prefecture, Japan) by three doctors trained in liver ultrasonography. Fatty liver was diagnosed in the presence of two of the three following criteria: increased hepatic echogenicity compared to the spleen or the kidney, blurring of liver vasculature and deep attenuation of the ultrasonographic signal. All subjects who had an abnormal liver on ultrasound were screened for hepatitis B and C (hepatitis B surface antigen [HBsAg] [Fujirebio, Tokyo, Japan] and anti-hepatitis C virus [Ortho Quick Chaser, third generation, c22-3, c200, NS5]). NAFLD was diagnosed in subjects who fulfilled ultrasonographic criteria for fatty liver, and who did not report alcohol consumption above the safe limit (< 14 units/week for men, < 7 units/week for females) and who were negative for hepatitis B and C markers.

### Statistical analysis

Data were entered in Epi Info 2000 (Centres for Disease Control and Prevention, Atlanta, GA, USA) and logical and random checks were done. Statistical analysis was done using SPSS version 16.0 (SPSS, Chicago, IL, USA). Continuous and categorical data were described using mean and standard deviations (SD) and percentages. Multivariate analysis was done using binary logistic regression. *P *< 0.05 was considered as significant.

## Results

Of the eligible 620 individuals in the two tea plantations, 403 participated in the study (response rate of 65%). Complete data were available in 401 participants (212 females). Almost all (99.3%) were either Indian Tamil (77.3%) or Sri Lankan Tamil (22%). About 74% were Hindus and 23% were Christians. Over 13% of the females had either a high waist circumference (WC) or a high Body Mass Index (BMI). Compared to females, a higher percentage of males (16%) had a high WC. Prevalence of NAFLD was 17.9% in this population. Mean age of those with NAFLD was 50.5 years (SD 7.9). Twice as many males were diagnosed as having NAFLD compared to females. The risk profile of the study subjects is given in Table 1. On bivariate analysis, male sex, higher education or income level, low or moderate levels of physical activity, BMI ≥ 25 kg/m^2^, WC ≥ 90 cm in males or ≥ 80 cm in females, systolic blood pressure of ≥ 130 mmHg, diastolic blood pressure of ≥ 85 mmHg, fasting plasma glucose of ≥ 5.6 mmol/L, insulin resistance (homeostasis model assessment of insulin resistance [HOMA-IR], > 1) and triglycerides ≥ 1.7 mmol/L were significantly associated with NAFLD (Table 2). On multivariate analysis, male sex, BMI, WC, diastolic blood pressure and plasma glucose were significant predictors of NAFLD. Those obese, defined either by high waist circumference or high body mass index, were approximately 5 times more likely to have NAFLD compared to non-obese individuals (Table 3).

**Table 1:** Risk profile of the study subjects

Variable	Males (*n *= 189) *N (%)*	Females (*n *= 212) *N (%)*	Total (*n *= 401) *N (%)*
BMI ≥ 25 kg/m^2^	19 (10.1)	28 (13.2)	47 (11.7)

High waist circumference^1^	30 (15.9)	29 (13.7)	59 (14.7)

Regular alcohol consumption^2^	103 (54.5)	16 (7.5)	119 (29.7)

Weekly alcohol consumption above safe limit^3^	34 (17.9)	6 (2.8)	40 (9.9)

Low/moderate activity levels^4^	46 (24.3)	45 (21.2)	91 (22.6)

High fasting plasma glucose^5^	60 (31.7)	39 (18.4)	99 (24.7)

Insulin resistance^6^	50 (26.5)	35 (16.5)	85 (21.1)

High systolic blood pressure^7^	115 (60.8)	117 (55.2)	232 (57.9)

High diastolic blood pressure^8^	93 (49.2)	93 (43.9)	186 (46.4)

High serum triglycerides^9^	83 (43.9)	56 (26.4)	139 (34.7)

Low serum HDL^10^	63 (33.3)	128 (60.4)	191 (47.6)

High ALT^11^	3 (1.6)	1 (0.5)	4 (0.9)

Fatty liver^12^	50 (26.5)	27 (12.7)	77 (19.2)

Prevalence of NAFLD	45 (23.8)	27 (12.7)	72 (17.9)

^1^waist circumference > 90 cm for men, > 80 cm for female, ^2^consumption of alcohol atleast once a week, ^3^> 14 units/week for men, > 7 units/week for females, ^4^as classified by International Physical Activity Questionnaire [IPAQ], ^5^fasting plasma glucose (FPG) ≥ 5.6 mmol/L or previously diagnosed type 2 diabetes, ^6^homeostasis model assessment of insulin resistance [HOMA-IR] > 1, ^7 ^systolic blood pressure ≥ 180 mmHg, ^8^diastolic blood pressure ≥ 85 mmHg, ^9^triglycerides ≥ 1.7 mmol/L, ^10^HDL (high-density-lipoprtein) < 1.03 mmol/L for males and < 1.29 mmol/L for females, ^11^ALT (alanine aminotransferase) > twice upper limit of normal, ^12^as determined by ultrasonographic criteria excluding excess alcohol users and those with viral infection.

**Table 2:** Factors significantly associated with NAFLD among the estate population

Variable	Number (%) with NAFLD	Odds Ratio	95% Confidence Interval
Male sex	45 (23.8)	2.1	1.2-3.7

Higher education level^1^	29 (40.8)	4.6	2.5-8.9

Higher income^2^	26 (31.7)	2.8	1.5-5.0

Low activity levels^3^	25 (27.5)	2.1	1.2-3.8

BMI ≥ 25 kg/m^2^	30 (63.8)	13.1	6.3-27.3

High waist circumference^4^	36 (61.0)	13.3	6.8-26.2

High systolic blood pressure^5^	52 (22.4)	2.2	1.2-3.9

High diastolic blood pressure^6^	49 (26.3)	2.9	1.7-5.3

High plasma glucose^7^	30 (30.3)	2.7	1.5-4.8

Insulin resistance^8^	37 (43.5)	6.2	3.4-11.2

High triglycerides^9^	39 (28.1)	2.7	1.6-4.7

^1^educated upto Ordinary Level (grade 10) or above, ^2^income > Rs.10,000 (~US$ 100), ^3^as classified by International Physical Activity Questionnaire [IPAQ], ^4^waist circumference > 90 cm for men, > 80 cm for female, ^5 ^systolic blood pressure ≥ 180 mmHg, ^6^diastolic blood pressure ≥ 85 mmHg, ^7^fasting plasma glucose (FPG) ≥5.6 mmol/L or previously diagnosed type 2 diabetes, ^8^homeostasis model assessment of insulin resistance [HOMA-IR] > 1, ^9^triglycerides ≥1.7 mmol/L

**Table 3:** Summary of logistic regression analysis of factors associated with NAFLD

Variable	β	Standard error	Odds ratio	95% Confidence interval	*P-*value
Male sex	0.919	0.334	2.6	1.3-4.8	0.006

BMI ≥ 25 kg/m^2^	1.576	0.506	4.8	1.8-13.0	0.002

High waist circumference^a^	1.664	0.444	5.3	2.2-12.6	< 0.001

High diastolic blood pressure^b^	0.779	0.320	2.2	1.2-4.1	0.015

High plasma glucose^c^	0.970	0.329	2.6	1.4-5.0	0.003

Constant	-3.430	0.363			

^a^waist circumference > 90 cm for men, > 80 cm for female, ^b^diastolic blood pressure ≥ 85 mmHg, ^c^fasting plasma glucose (FPG) ≥ 5.6 mmol/L

## Discussion

Our study found a NAFLD prevalence of nearly 18% despite this Indian Tamil ethnic population being from a rural estate area of Sri Lanka with wages earned mainly through manual labour, and where limited access to food, sanitation and transport still exists. Like in other studies, NAFLD was associated with constituent features of the metabolic syndrome. Although much lower than the 33% prevalence found in a community based study in a relatively sedentary, urban Sri Lankan population [5], the figure is well within the range of 16% to 23% identified by other population based studies that have used ultrasonagraphy for diagnosis of NAFLD [9,10]. A prevalence of NAFLD similar to our study has been reported from Hong Kong, Korea and Malaysia [10]. However, unlike in our population, access to excessive amounts of food and low activity levels were important features in these studies. Thus, our study strengthens the existing evidence for Asian Indians' increased predisposition to visceral fat accumulation; a feature that may be present from birth [11,12].

Although liver biopsy remains the gold standard to confirm diagnosis of NAFLD, this is impractical in epidemiological studies. Non-invasiveness and low cost of ultrasonography makes it the most acceptable technique to diagnose fatty liver in field epidemiological studies. High sensitivity for diagnosing fatty liver has been reported using imaging techniques, including ultrasonography [13]. The ultrsonographic criteria we used to detect fatty liver have an adequate threshold for detection of steatosis when more than 33% of hepatocytes contain fat on liver histology [14]. Exclusion of those with evidence of hepatitis B and C infections and an alcohol intake above the safe limits are also prerequisites to diagnose NAFLD [15]. One limitation of our study was obtaining information on alcohol consumption only by direct questioning of the subject. Another was that we were unable to assess inter-observer reliability between the sonographers before the commencement of the study.

Male sex was a significant independent predictor of NAFLD in our study. Though early studies in Western populations found that NAFLD was more prevalent in women, a more recent population-based study from the USA has shown that men have a higher prevalence of presumed NAFLD, based on elevated aminotransferases [16]. Another recent study from Korea also showed a higher prevalence of NAFLD among men compared to women [17]. Undisclosed alcohol abuse, which has been given as one possible explanation for the higher prevalence of NAFLD in men, may be relevant in this study too. 18% of men reporting weekly alcohol consumption above safe limit is one indication for this. However, it is also known that women in the estate sector in Sri Lanka are more physically active compared to the estate males; 65% of the plantation labour force is constituted of female tea pluckers [18]. Abdominal obesity which is considered to be an important risk factor for metabolic syndrome and related diseases was also more prevalent in estate males than females, though not significantly. These are possible explanations for finding male sex as an independent risk factor for NAFLD in the present study.

There is increasing evidence for a genetic basis for the development of NAFLD. Schwimmer et al. report familial factors as a major determinant of NAFLD [19]. Others have reported racial and ethnic differences in the prevalence of the condition [20,21]. Petersen et al. have shown that polymorphisms C-482T and T-455C in *APOC3 *are associated with nonalcoholic fatty liver disease and insulin resistance in healthy Asian Indian men [22]. Our finding of a high NAFLD prevalence in a rural, physically active, predominantly Tamil estate population from Sri Lanka supports results of studies reporting ethnic variations in susceptibility.

## Conclusion

In conclusion, we found that nearly one in five people in a predominantly Indian Tamil, rural, physically active, economically deprived population had NAFLD. The condition was associated with constituent features of the metabolic syndrome. Our findings also support results of family studies and studies reporting ethnic variations in susceptibility which suggest that genetic factors can also play an important role in determining disease risk.

## List of abbreviations

NAFLD: Non-alcoholic fatty liver disease; BMI: Body mass index; WC: Waist circumference.

## Competing interests

The authors declare that they have no competing interests.

## Authors' contributions

HJD, ARW, ASD, MJP & NK were involved in conceptualizing the study. ASD, SR, & UK were involved in data gathering. MJP was involved in statistical analysis and writing the manuscript. All authors read and approved the final manuscript and helped in editing the final copy.
